# Roles of Motor Cortex Neuron Classes in Reach-Related Modulation for Hemiparkinsonian Rats

**DOI:** 10.3389/fnins.2021.645849

**Published:** 2021-04-27

**Authors:** Min Li, Xuenan Wang, Xiaomeng Yao, Xiaojun Wang, Feiyu Chen, Xiao Zhang, Shuang Sun, Feng He, Qingmei Jia, Mengnan Guo, Dadian Chen, Yue Sun, Yuchuan Li, Qin He, Zhiwei Zhu, Min Wang

**Affiliations:** ^1^Key Laboratory of Animal Resistance Biology of Shandong Province, College of Life Science, Shandong Normal University, Jinan, China; ^2^Shanghai Mental Health Center, Shanghai Jiaotong University School of Medicine, Shanghai, China; ^3^School of Nursing, Qilu Institute of Technology, Jinan, China; ^4^The First Hospital Affiliated With Shandong First Medicine University, Jinan, China; ^5^School of International Education, Qilu University of Technology, Jinan, China; ^6^Editorial Department of Journal of Shandong Jianzhu University, Jinan, China

**Keywords:** Parkinson’s disease, motor cortex, microelectrode, spike, local field potential, rats, hemi-parkinsonian, 6-OHDA

## Abstract

Disruption of the function of the primary motor cortex (M1) is thought to play a critical role in motor dysfunction in Parkinson’s disease (PD). Detailed information regarding the specific aspects of M1 circuits that become abnormal is lacking. We recorded single units and local field potentials (LFPs) of M1 neurons in unilateral 6-hydroxydopamine (6-OHDA) lesion rats and control rats to assess the impact of dopamine (DA) cell loss during rest and a forelimb reaching task. Our results indicated that M1 neurons can be classified into two groups (putative pyramidal neurons and putative interneurons) and that 6-OHDA could modify the activity of different M1 subpopulations to a large extent. Reduced activation of putative pyramidal neurons during inattentive rest and reaching was observed. In addition, 6-OHDA intoxication was associated with an increase in certain LFP frequencies, especially those in the beta range (broadly defined here as any frequency between 12 and 35 Hz), which become pathologically exaggerated throughout cortico-basal ganglia circuits after dopamine depletion. Furthermore, assessment of different spike-LFP coupling parameters revealed that the putative pyramidal neurons were particularly prone to being phase-locked to ongoing cortical oscillations at 12–35 Hz during reaching. Conversely, putative interneurons were neither hypoactive nor synchronized to ongoing cortical oscillations. These data collectively demonstrate a neuron type-selective alteration in the M1 in hemiparkinsonian rats. These alterations hamper the ability of the M1 to contribute to motor conduction and are likely some of the main contributors to motor impairments in PD.

## Introduction

Many studies have been conducted to understand the correlations between primary motor cortex (M1) neuron activity and voluntary movement in Parkinson’s disease (PD), since basal ganglia circuits are closely tied to the cortex (Reiner et al., 2010; Wu et al., 2011; Quiroga-Varela et al., 2013). It is likely that prominent alterations in neuronal discharge patterns in the basal ganglia also arise in the cortex (Walters et al., 2007; Brazhnik et al., 2012, 2014; Li et al., 2012; Devergnas et al., 2014; Valencia et al., 2014). Direct dopaminergic denervation of M1 may also contribute to abnormal voluntary movements in PD (MacDonald and Halliday, 2002; Braak et al., 2003, 2006; Lindenbach and Bishop, 2013). It has been reported that deficits in the M1 are associated with PD pathophysiology in PD patients and in animal models of PD (Parr-Brownlie and Hyland, 2005; Pasquereau and Turner, 2011; Li et al., 2012; de Hemptinne et al., 2013; Pasquereau and Turner, 2013). PD symptoms are accompanied by certain alterations, including abnormal firing rates and patterns, pathologic oscillatory activity, and increased synchronization throughout basal ganglia-cortical circuits (Walters et al., 2007; Ellens and Leventhal, 2013; Devergnas et al., 2014; Valencia et al., 2014; Galvan et al., 2015; Wang et al., 2015; Dupre et al., 2016). However, the available data are inconsistent regarding whether there is a decrease or an increase in the movement-related activity of M1 neurons (Parr-Brownlie and Hyland, 2005; Pasquereau and Turner, 2011; Pasquereau and Turner, 2013), and some studies have reported an increase (Lefaucheur, 2005) or no change in cortical neuron activity (Goldberg et al., 2002).

In addition, dopamine depletion is correlated with an alteration in certain local field potential (LFP) frequencies, especially those in the beta range (broadly defined here as any frequency between 12 and 35 Hz), and has been reported to occur in the basal ganglia and cortex in patients and PD animal models (Brazhnik et al., 2012; Devergnas et al., 2014; Wang et al., 2015; Vitrac and Benoit-Marand, 2017; Holt et al., 2019). LFPs are obtained by low-pass filtering extracellular field potentials and reflect different neural processes, including integrative synaptic dynamics, that cannot be captured by measuring the spiking activity of a few neurons (Buzsaki et al., 2012). Therefore, LFPs provide a unique perspective on key integrative synaptic processes in cortical populations (Debeir et al., 2005; Sharott et al., 2005; Pasquereau et al., 2019). There is considerable evidence that changes in firing rates and LFPs in the M1 play an important role and lead to dynamic remodeling of movement representation in parkinsonism (Goldberg et al., 2004; Pasquereau and Turner, 2011; Brazhnik et al., 2012; Li et al., 2012, 2019; Valencia et al., 2014; Pasquereau et al., 2016; Swann et al., 2016). Furthermore, spike and LFP synchronization have been increasingly used to explain the pathophysiology in the parkinsonian brain (Vitrac and Benoit-Marand, 2017; Holt et al., 2019). Distinguishing changes in simultaneously recorded LFPs and spikes is therefore crucial for delineating the neuronal activity that leads to parkinsonian symptoms and for determining the role of these activities in healthy processing (Debeir et al., 2005; Pasquereau et al., 2016; Pesaran et al., 2018). Despite the implication of the M1 in PD pathophysiology, the distinct contributions of specific types of neurons to pathophysiological information processing during different behaviors in PD are currently unclear (Goldberg et al., 2002; Parr-Brownlie and Hyland, 2005; Pasquereau and Turner, 2011; Brazhnik et al., 2012; Nambu et al., 2015). Few studies have simultaneously investigated both single and local population activity in the M1 of 6-OHDA-intoxicated rats (Brazhnik et al., 2012; Dejean et al., 2012; Pasquereau et al., 2019).

In this study, we addressed these questions by characterizing the differential alteration in neuronal activity within subpopulations in the M1, focusing on interneurons and projection neurons in layer 5, that contribute to parkinsonism. The experiments described rely on behavioral training and electrophysiological recordings to study voluntary limb movement deficits and related abnormalities of M1 neuron extracellular electrical activity in the context of unilateral dopamine depletion. Using multichannel recording arrays implanted in layer 5 of the M1, we recorded extracellular single-neuron spiking activity and LFPs in control and unilateral 6-hydroxydopamine (6-OHDA-treated) rats during rest and skilled reach-to-grasp movements. This forelimb reaching task has been shown to have limited usefulness in assessing the generation and execution of voluntary limb movements, such as in chronic animal models of Parkinson’s disease (Parr-Brownlie and Hyland, 2005; Pasquereau et al., 2016; Zanos et al., 2018; Holt et al., 2019). This task was used in our previous study, and we found that the rats were able to successfully perform the reach-to-grasp task with one forelimb and exhibited characteristics of 6-OHDA-induced modifications in spiking and LFPs in the thalamic parafascicular nucleus (Wang et al., 2016). The use of this task could provide direct information about several key issues regarding the involvement of M1 electrical activity at the single-cell and population levels and could provide insight into which particular aspects of M1 function are abnormal in parkinsonism.

## Materials and Methods

### Animals

All experimental procedures were performed according to the National Institutes of Health Guidelines for the Care and Use of Laboratory Animals and approved by the Animal Ethics Committee of Shandong Normal University (Protocol Number: AEECSDNU2018015, 2018). Adult male Wistar rats weighing 280–320 g (Shandong University, China) were used for the experiments. Male Wistar rats were housed at 22–23°C on a 14: 10 h light/dark cycle. The animals had free access to water, but food intake was limited to maintain motivation for obtaining food with their forepaws. Every effort was made to minimize the number of animals used and the pain they experienced. We anesthetized the animals using urethane (1.0 g/kg) and administered carprofen (5 mg/kg) as an analgesic.

### Behavioral Training and Monitoring

**FIGURE 1:**
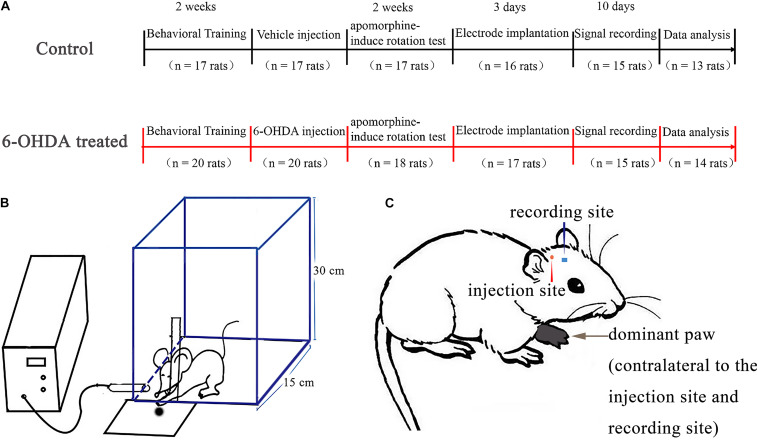
Experimental timeline and design. In all experiments, all rats (*n* = 37) underwent behavioral training and monitoring in a rectangle transparent Plexiglas box and the most frequently used paw was determined as dominant paw. After 14 days, one group (6-OHDA-treated, *n* = 20 rats) was given a unilateral 6-OHDA treated in the medial forebrain bundle (MFB) of the hemisphere contralateral to the dominant paw, the other group (control, *n* = 17 rats) was given saline. After 14 days, lesion efficacy was tested by apomorphine-induced rotation test. Rats from the first group (6-OHDA-treated, *n* = 14 rats) with successful lesioned effects and the second group (control, *n* = 13 rats) underwent surgery to implant recording electrodes in the motor cortex of the same hemisphere as the injection side. Signal recording began 3 days after the electrodes were implanted. Rats were placed in the reaching box in order to record the neuronal activity at both rest and reaching periods. **(A)** Illustration of the self-made chamber design to reach for food pellets. Test chamber used to assess trained reaching to catch food, and this schematic shows that the rat could reach through a slot to make contact with food from the tray. This reach would simultaneously create a mark on the computer via the infrared obstacle avoidance sensor attached to the chamber front wall **(B)**. A rat showing naming conventions. This example is a rat whose left paw dominates after food reaching training. Intracerebral injections of the vehicle or 6-OHDA (in MFB) and recording electrode (in M1) are contralateral to the dominant paw **(C)**.

We used the same apparatus to assess the reaching performance of the rats as in a previous study (Wang et al., 2016) (Figure 1A). Before training, rats were restricted to 15 g of standard rat chow (ke ao xie li company, Beijing, China) per day to ensure that they were motivated to execute the reaching task to obtain palatable food (Parr-Brownlie and Hyland, 2005). The experimental chamber (self-made, Figure 1B) was a transparent rectangular Plexiglas box (20 × 15 × 30 cm in size). A vertical slit (1.5 cm wide) was made in the front wall of the experimental box to train the animals to obtain food using their forepaws. A square plate containing food pellets (grinding standard rat chow) was mounted in front of the slit outside the experimental chamber. All rats (*n* = 37) were trained to reach through the slit and retrieve food pellets. Every training day consisted of three training sessions; every session consisted of 30 trials. One trial was defined as one continuous reaching which started from approaching the slit and grabbing the food, and ended with retracting their paw. A daily 30 trial training session took around 6–10 min and the rats took an hour break between every session. The training section lasted 2 weeks before the surgery. The preferred paw was defined as the paw used for 70% of reaches in training (Elena et al., 1994; Parr-Brownlie and Hyland, 2005; Bosch-Bouju et al., 2014; Guo et al., 2015). All rats included in our research demonstrated a paw preference when training. Once the dominant paw was defined, the pellets were placed in front of the test chamber on the contralateral side of the dominant paw to prevent the simultaneous use of both paws. Animal behavior was evaluated by assessing successful reaches, which were defined as reaching for, grabbing, and retrieving of a food pellet with the preferred paw. The rats required 3–5 days to learn to reach for food successfully, and the task was performed for another 7–10 days to demonstrate the stability of paw preference. The animals adopted a suitable posture to reach their paws through the slit, and each set of reaching trials was performed with the body in a similar posture and orientation. Therefore, after a rat made a successful reach, there was a short pause before the next food pellet was presented by the experimenter to ensure that the rat was able to reposition itself at the food aperture for the next food pellet. The success rate was calculated as the percentage of successful reaches relative to total reach attempts. Only the rats that displayed a paw preference (also called dominant paw, Figure 1C) and reliably reached were included in the experiments. Supplementary Video 1 shows the rats in the control group practicing fetching food using their forepaw, Supplementary Video 2 shows the rats in the 6-OHDA group practicing fetching food using their forepaw.

A digital video camera (Logitech AF, Taiwan) was used to record the trajectory of the animals’ paws during the food-reaching process (30 frames/s) for further offline analysis with the concurrently generated neuronal signals. An infrared obstacle sensor (E18-D80NK, Chengdu), which included an infrared light beam inductor linked with a multichannel recorder (The OmniPlex D, Plexon Inc.) by a digital input-output interfacing, was used to provide a trigger signal (Figure 1C). When the infrared light beam was interrupted, the system converted the changed voltage in transistor-transistor logic (TTL) pulses to digital input and generated a sign on the recording computer screen interface. Therefore, when an animal paw passed through the slit in the front wall of the transparent Plexiglas chamber and reached to grasp food, the infrared beam was interrupted, and the system produced a mark in the ongoing neural activity recording on the computer screen. The reach-related movement modulations in each trial were derived around the mark produced by the infrared trigger signal.

CamStudio screen-recording video software (MicroImages, Inc., United States) was used to continuously record task performance in real-time and record simultaneous neural activity through interaction of the computer with the multichannel recording system (The OmniPlex D, Plexon, Inc.) during all recording sessions. Based on the screen recordings and offline frame-by-frame analysis, we were able to construct a peri-event analysis time window. Each trial was identified in detail with behavioral tracking and the synchronization of neural signals.

### Unilateral 6-Hydroxydopamine Lesion

After the training period, the rats were randomly divided into two groups. One group (the 6-OHDA treated group, *n* = 20) was administered a unilateral injection of 6-OHDA into the medial forebrain bundle (MFB, contralateral to the dominant paw, Figure 1C). The other group (the control group, *n* = 17) was administered a unilateral injection of the same volume of vehicle (0.02% ascorbic acid in physiological saline) into the MFB (contralateral to the dominant paw, Figure 1C). Unilateral 6-OHDA-induced depletion of dopamine in the dopamine nigrostriatal pathway was induced as previously described (Geng et al., 2016a; Wang et al., 2016). The rats were fully anesthetized with urethane (1.0 g/kg i.p., Sigma) until they were completely unresponsive to painful stimuli (firm toe or tail pinch), and additional 10% doses were administered as needed. The animals were then placed in a stereotaxic frame (Reiwode 68001, Shenzheng, China) on a heating pad to maintain body temperature at 37 ± 0.5°C. The skull was exposed above the MFB at the following stereotaxic coordinates: AP –2.16 mm, ML 2.1 mm (the injection side contralateral to the dominant paw, Figure 1C), and DV 8.5 ± 0.1 mm (Paxinos and Watson, 2007). The rats were injected with desmethylimipramine (15 mg/kg, i.p.) 30 min prior to the intracerebral infusion to protect noradrenergic neurons (Wang et al., 2016). A hole was drilled in the skull on the side contralateral to the preferred paw, as determined via behavioral training. At a rate of 1 μl/min, 2 μg/μl 6-OHDA in 3 μl of 0.9% saline and 0.02% ascorbic acid was injected via a microsyringe into the MFB. The control rats only received the same volume of vehicle at the same coordinates. The microsyringe was kept at the injection site for another 5 min to prevent neurotoxin diffusion. Finally, the incision was sutured, and the long-acting analgesic carprofen (5 mg/kg, Sigma) was injected subcutaneously. The animals were kept on a heated platform at 37°C until they had fully recovered from anesthesia. Then, the animals were returned to their cages and housed individually. The rats received postoperative care and were checked every day for 1 week after the surgery.

The extent of the dopaminergic lesion was evaluated two weeks after surgery by apomorphine-induced rotation test (0.05 mg/kg, s.c.) (Yuan et al., 2004). In order to reduce the interference to our electrophysiological signals, an automated rotameter device was not used to count the number of rotations. We directly captured the video of apomorphine-induced rotational test. Two people blind to the test were responsible for the subsequent manual analysis. Supplementary Table 1 shows the values of the apomorphine-induced rotation tests in the control and 6-OHDA groups. Supplementary Video 3 shows the example of the apomorphine-induce rotation tests of the control group, while Supplementary Video 4 shows the example of the apomorphine-induce rotation tests of the 6-OHDA group. Surgery was considered successful in animals that performed more than seven (contralateral rotations/min) contralateral rotations to the 6-OHDA-treated site within 20 min. Only these animals, which were considered the 6-OHDA-treated group, were used for behavior tests and electrophysiological recordings. After completing all experiments, lesions were further assessed post-mortem using immunohistochemistry for tyrosine hydroxylase.

### Recording Electrode Implantation

Two weeks after the 6-OHDA or sham injection, a microwire array of extracellular recording electrodes was implanted into the M1 contralateral to the preferred reaching paw (i.e., the hemisphere ipsilateral to 6-OHDA or vehicle injection) in each rat. The implanted microwire electrode array was manufactured from eight nickel–chromium and HFV natural-insulated microwires (12.5 μm in diameter, California Fine Wire, United States) and was arranged in a 2 × 4 pattern, with interelectrode intervals of 100 μm between each microwire spaced 0.25 × 1.0 mm^2^ apart (i.e., 0.25 × 1.0 mm^2^). The size of the entire electrode array was described in detail in our previous study (Wang et al., 2016; Zhang et al., 2019).

In addition, uninsulated silver wires (125 μm) served as ground wires. Electrode implantation was performed under urethane (1.0 g/kg i.p., Sigma-Aldrich, additional 10% doses as needed) anesthesia following the same general protocol as that used for 6-OHDA and sham injection. A craniotomy was made in the cranium above layer V the area of the M1 representing the unilateral forelimb at the following coordinates, as determined by the atlas of Paxinos and Watson: AP + 1.0 mm, ML 2.0 mm (contralateral to the dominant paw, Figure 1C), DV 1.5–2.5 mm (Paxinos and Watson, 2007). During electrode array implantation, spike activity was continuously monitored to help locate the electrode tip at the targeting position in each rat. Along the trajectory to the M1, there was a relatively silent area with a low-amplitude background, followed by layer 3 (1.0–1.5 mm deep from the skull surface). Neurons in layer 3 typically had sparse lower amplitude spikes, which represented the firing activity amplitudes of discrete neurons. When the electrode arrays were advanced to layer 5 (2.0–2.5 mm deep from the skull surface), there were relatively high amplitude spikes with an irregular firing pattern and discrete spike bursts in rapid succession. After reaching the final targeting layer 5, the ground wires were wrapped around stainless-steel screws that were anchored to the surface of the cranium. The electrode array was anchored to the surface of the skull with metal screws and secured with dental cement. After the operation, the rats were given the analgesic carprofen (5 mg/kg, Sigma) and postoperative care according to the same general protocol as that used for 6-OHDA and vehicle injection.

### Electrophysiological Data Acquisition

Both extracellular single-unit and LFP recordings were conducted for 1 week after microwire electrode implantation (i.e., 3 weeks after 6-OHDA or vehicle injection). Only high-quality isolated single unit and LFP recording data obtained following stabilization 2–3 weeks post-implantation were subjected to further offline analysis. During recording sessions, signals from the electrodes were amplified and filtered using the multichannel recording system (The OmniPlex D, Plexon Inc.). Channels with distinguishable single-cell activity and LFPs were recorded using offline sorter version 4 (Plexon, Inc. Dallas, United States) data acquisition analysis software. For each channel recording, activity on the ground wire served as the reference signal for all other wires. Channels with distinguishable cell activities were amplified 2000×, bandpass filtered at 300-8 kHz and sampled at 40 kHz. The LFP recordings were bandpass filtered at 0.5–200 Hz and sampled at 1 kHz. A 50 Hz notch filter was employed to reduce line artifacts (Aton et al., 2014; Wang et al., 2016; Jahn et al., 2020).

An amplitude threshold was set using the sorting software to exclude most background noise, spike waveforms were visualized, raster plots were generated, and waveform shape template matching was performed. Only spikes exceeding this threshold were stored. LFPs were simultaneously recorded without non-stationary periodic noise and baseline drift. For overall measures of neuronal activity, a 30 min epoch of simultaneously recorded spiking activity and LFPs free of artifacts from every recorded neuron during rest and movement were used.

### Identification and Classification of Neurons

We used the same methods to detect and sort spikes from the saved raw recording data as those used in previous studies (Nicolelis et al., 2003; Dodson et al., 2016; Geng et al., 2016b; Wang et al., 2016). The first step was setting the voltage threshold for each of the channels processing signals from the implanted microwire electrodes. All signals that crossed the set voltage threshold were defined as unsorted waveforms and were subjected to subsequent spike sorting analysis with Offline Sorter (Plexon, Inc., Dallas, United States). The putative single-unit activity waveforms were isolated with standard “spike sorting” procedures, including template matching, principal component analysis (PCA), and supervised clustering. Clusters of points observed in the PC space were inspected, and the experimenter visualized the waveform associated with each point, measured the waveform’s trough-to-peak duration (between the first negative deflection and the peak of the second positive deflection of a spike waveform), and selected a group of points to set up interspike interval (ISI) histograms. Next, based on multiple parameters (waveform voltage, waveform shape, clusters, ISIs, etc.), the experimenter then manually eliminated the electrical noise and artifacts and defined the clusters shown to contain waveforms similar to those of the sorted waveforms. In addition, the sorted single units had to exhibit a clearly recognizable refractory period (>2 ms) in their ISI histograms and a distant trough-to-peak duration when compared with other neuronal waveforms. Of the individual parameters, the trough-to-peak measure was easily detectable for spike duration and provided the most reliable separation of two classes (Barthó et al., 2004). These allowed excellent separations of waveforms collected from single or multiple electrodes into distinct sorts.

### Analysis of Spike Firing Rate and Pattern

The classified spiking properties and functional activity in relation to behavioral task performance (rest or movement) were analyzed. The recording data were imported into NeuroExplorer version 5 (NEX Technologies, NC) to extract spike timestamps for resting behavior and PETH movement. The mean firing rate was calculated by dividing the total number of recorded spikes by the total time. We also computed the coefficient of variance (CV) of the ISIs and the mode and asymmetry index of ISIs, which served as the indexes of the firing pattern and described the extent of the regularity of neuronal discharge (Gao et al., 2019; Jacob et al., 2020). The CV was computed for each recording as a measure of the regularity of the spike firing defined as the standard deviation divided by the mean ISI. Exponential distributions have a CV of one, i.e., they describe more irregular discharge patterns, whereas distributions derived from more regular ISIs have CV values below one with an approximately Poisson distribution (Alam et al., 2012; Geng et al., 2016b). The “mode” resembled the most frequent ISI. The asymmetry index represented the ratio of the mode to the mean ISI. It provided information regarding the shape of the ISI histogram or the regularity of the discharge pattern. An asymmetry index close to one indicated that there was a relatively regular firing pattern. An index < 1 reflected an asymmetrical shape, which indicated an irregular firing pattern (Alam et al., 2012, 2015).

### Behavioral-Related Neuronal Activity

Next, through direct observation and video camera recording, we performed two types of analyses to characterize M1 extracellular electrical activity corresponding to rest or movement. First, we analyzed the firing properties and LFPs of neurons when rats were at rest, which was defined as when they were awake but not attentive to their environment (i.e., when the rats were not whisking or looking around but had their eyes open). The mean firing rate or LFP spectral power was determined as the total number of recorded data divided by the total time. Second, we analyzed movement-related modulations in mean firing rate and LFPs; movements were defined as those involving forelimb movement (determined from video recordings), i.e., with a duration associated with each successful reach (i.e., when the rats were successful in using their preferred paw to reliably stretch out of the slit in the front wall of the test chamber and acquire a food pellet).

For analyses of reach-related modulations, the reach-related firing rates were derived from average peri-event raster plots and peri-event time histograms (PETHs) centered on the interruption of the infrared beam (i.e., marking every reach into the slit and the grasp phase of each reaching movement) (Hyland and Jordan, 1997; Parr-Brownlie and Hyland, 2005; Bosch-Bouju et al., 2014; Wang et al., 2016). The time stamps of spike activity during task performance were exported to NeuroExplorer software (Nex Technologies, United States), and peri-event raster plots and PETHs (bin width of 5 ms, smoothed with a sliding five-point Gaussian window) were used to calculate the mean spiking rate of task performance to detect and quantify reach-related modulations in firing rate and to provide a visual display. The firing rates in PETHs were derived from the center on the marker of breakdown of the infrared beam (i.e., indicating the time the rat took to reach out with the preferred paw, while the recordings online were indicated by a mark on the recording computer screen interface) (Putrino et al., 2011). In addition, we were also able to analyze the trajectory of the animals’ paws offline frame by frame in detail through CamStudio screen-recording software and set up time windows for each successful reach (i.e., we confirmed the time windows for reaching, grabbing, and retrieving a food pellet). Therefore, movement periods of PETHs were determined using annotated recordings with keystrokes offline to indicate movement onset marked when the rat began to lift its paw (‘time = 0’ in PETHs) and the movement end time marked when the rat completed the reach. The spikes were recorded across all trials (PETHs) and were constructed around the beam interruptions and between the two manual movement marked signs to ensure that each successful reach movement was covered. Only neurons recorded during the spontaneous execution of such movement periods were considered for further analysis of movement-related firing. For each PETH, the baseline was defined as the average spikes/second during the period –0.6 s before the time window of the PETH, and the short time duration provided a behavioral clamping stage to ensure that the rats maintained a similar behavioral state. Task performance was considered significantly modulated if the neural firing rate was significantly modulated during the stages of task performance compared with the “background” (baseline activity) periods.

### Analysis of Local Field Potentials

Spectral analysis of LFPs was conducted using the open-source data analysis toolbox Chronux 2.0 and MATLAB 2010a (Mathworks, Natick, MA, United States) signal processing toolbox functions along with custom software. The actual spectral data analysis was estimated by the Chronux function mtspectrumc computed using a multitaper method, with five tapers, a time bandwidth product of 3, a window width of 0.5 s, and a sampling frequency of 1000 (Masimore et al., 2004). To examine frequency band-specific characteristics, as in our previous studies, the relevant LFP power spectra were analyzed in bands of 0.7–12, 12–35, 35–70, 70–100, and 100–200 Hz, respectively, as described previously (Wang et al., 2016). We calculated each band range power as a function to give a ratio across the entire signal power (1–200 Hz), which was expressed as the relevant power of total power to overcome influences due to individual non-specific differences in absolute power. To visualize spectral power changes over time for the selected epochs, we use the scripts of the time-frequency toolbox, which are available at http://tftb.nongnu/org, to calculate the spectra using a short-term Fourier transform (Kuhn et al., 2008). The spectra of the short-term Fourier transform were scaled to best show the enhanced LFP resolution with minimum background noise (Sharott et al., 2009; Baker et al., 2014).

### Analysis of the Relationship Between Spikes and LFPs

#### Spike-Field Coherence Estimation

To estimate the relationships between spike timing and the ongoing LFPs at different frequencies and over time, spike-field coherence estimation was performed using a multitaper method implemented in Chronux 2.12^1^ and MATLAB 2010 a (Mathworks, Natick, MA, United States). The coherence value was computed using the Chronux coherencycpt function (Bokil et al., 2010) using LFPs at 0.7–12, 12–35, and 35–70 Hz and the corresponding spikes. These frequency ranges were chosen according to our computed LFP, which indicated altered mean power frequency distributions between depleted and control rats. Coherence measures the linear association between two signals, which are used as an indicator of the coherence value between spiking and LFPs in the frequency-domain analysis and vary from 0 (no coherence) to 1 (absolute coherence) (Sharott et al., 2005; Hammond et al., 2007; Porter et al., 2020).

#### Spike-Field Phase Estimation

The instantaneous phase relationships between M1 spikes and LFPs in specific frequency bands were analyzed by MATLAB and functions of the Circular Statistics toolbox (Berens, 2009; Sharott et al., 2012; Abdi et al., 2015). LFPs were first bandpass-filtered (using a three-order Butterworth filter) before a Hilbert transform was applied to analyze the instantaneous phase relationships between M1 spike times and LFP frequency bands at 0.7–12, 12–35, and 35–70 Hz (Nakamura et al., 2014; Sharott et al., 2017). In this formulation, peaks in the specific frequency LFP oscillations correspond to a phase of 0° and troughs to a phase of 180° (Klausberger et al., 2003). Accordingly, circular phase histogram plots and circular statistical descriptions were calculated using the phase values for each spike in the Circular Statistics toolbox. Among the entire analyzed epoch (i.e., a firing of at least 40 spikes, a sampling criterion to ensure accurate determination of circular means and the significance of any phase-locked firing), the phase distribution of each neuron was tested for uniformity with a Rayleigh test (*p* < 0.05) to identify significant modulation of individual neuron spiking by LFPs in different frequency bands. Subsequently, neurons that were defined as phase-locked firing in the LFP-specific frequency bands could be used for the calculation of mean phase angles and vector lengths and for further statistical comparisons between the different groups. The mean vector length that was used to quantify the level of phase locking around the mean phase for individual neurons (computed using the angles of each spike) and for populations of neurons (computed using the mean angle for each neuron) varies between zero and one. The closer to one, the more concentrated the angle distribution around the mean phase angle, indicating that the neuron is more correlated with the LFPs (Womelsdorf et al., 2007; Sharott et al., 2012; Abdi et al., 2015). In the plotted rose circular histogram, we used a line radiating from the center to show the vector and length of the mean phase angle (Walters et al., 2007; Escande et al., 2016; Zhang et al., 2019).

### Histology and Immunochemistry

**FIGURE 2:**
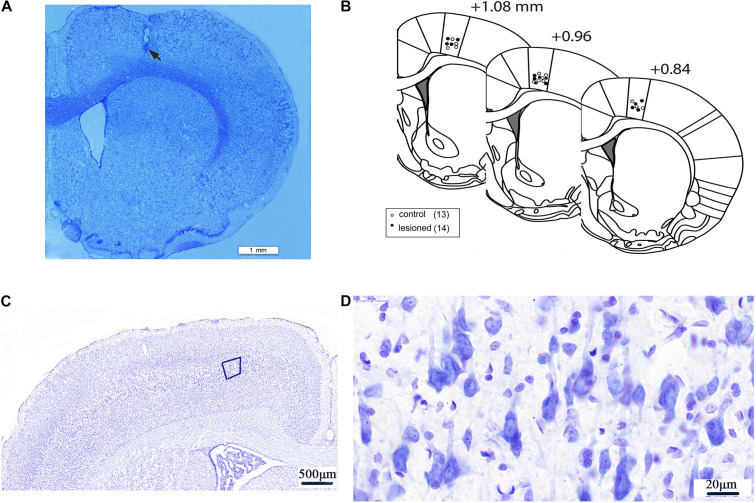
Electrode recording sites and neurons in layer 5 of M1. An example of low magnification (20×) coronal section of a rat brain displaying the histological verification of the recording electrode tip at layer V of the M1 by Nissl staining. Scale bar = 1 mm. The electrode tip was marked by electrolytic lesion (black arrow) **(A)**. Three schematic drawings of the rat coronal brain sections were adapted from the rat brain atlas (Paxinos and Watson, 2007), indicating the recording electrode sites in the M1 of 6-OHDA-treated rats (black circles) and control rats (white circles) **(B)**. Neurons in Layer 5 of M1) (50×) **(C)**. The magnification of the fifth layer of M1) (1250×) **(D)**.

Following the completion of recordings, rats were deeply anesthetized by overdose of urethane (3 g/kg, i.p.). The tips of the recording electrodes were passed through a positive electrical current (10 μA, 10 s × 3 times) through the 2–3 microwires of the electrode to mark the M1 recording site. The rats were then perfused intracardially through the heart with 4% paraformaldehyde mixed with 1% potassium ferricyanide in phosphate-buffered saline (PBS). The rats were decapitated, and the brains were post-fixed in 4% paraformaldehyde solution overnight and subsequently immersed in 30% sucrose in PBS solution for 48 h at 4°C until they descended to the bottom of the glass bottle. Sequential coronal sections (40 μm in thickness) at the position of 0.84–1.08 mm anterior to the bregma were cut using freezing microtome (CRYOSTAR NX50, Thermo Fisher, United States) (Paxinos and Watson, 2007). Next, each of the sections was stained with Nissl for verification of electrode recording placement. Only rats with correct electrode placement in layer 5 of the M1 were included for data analysis (Figures 2A–D).

Qualitative assessment of 6-OHDA-induced lesions in the substantia nigra pars compacta was conducted post-mortem via immunohistochemical analysis of tyrosine hydroxylase. Twelve 40-μm-thick coronal sections per animal were cut through the midbrain (Bregma –4.80 to –5.28 mm) for tyrosine hydroxylase (TH) immunohistochemistry to assess and evaluate the extent of dopamine (DA) cell lesions (Paxinos and Watson, 2007). The prepared coronal sections containing the substantia nigra were washed three times with 0.01 M PBS for 5 min and then incubated with 0.01 M PBS (pH 7.4) containing 0.5% Triton X-100 and 5% normal donkey serum for 6 h at the room temperature. The blocking solution was removed before the sections were incubated with primary antibody solution (rabbit polyclonal anti-TH antibody; 1: 1000; ab6211; Abcam Inc., Cambridge, United Kingdom) overnight at 4°C. The next day, the sections were rinsed with 0.2% Triton-PBS solution and were further incubated with a secondary antibody (FITC fluorescent dye (green) -conjugated donkey anti-rabbit IgG; 1: 200; sc-2090; Santa Cruz, United States) for 2 h at room temperature. Then, the sections were washed several times in 0.01 M PBS (4 × 10 min), attached to gelatin-coated slides, dehydrated in the air, then dipped in glycerine (containing 20 mg/ml 1,4-diazabicyclo octane) (Sigma, United States) and 10% TrisHcl (0.2 mol/L, pH 7.4). Coverslips were applied and fixed with nail polish. All serial sections were stored at –20°C and prepared for fluorescence microscopy (TCS SPE; Leica, Germany).

The morphology of the substantia nigra pars compacta was observed, and TH-positive neurons in this region were imaged using a computer interfaced with a laser scanning confocal microscope (TCS SPE; Leica, Germany) and saved in LAS AF series 009 software (Leica, Germany). The quantity of TH-positive cells was analyzed by QuPath software and an average for each section for each animal was determined (Bankhead et al., 2017).

**FIGURE 3:**
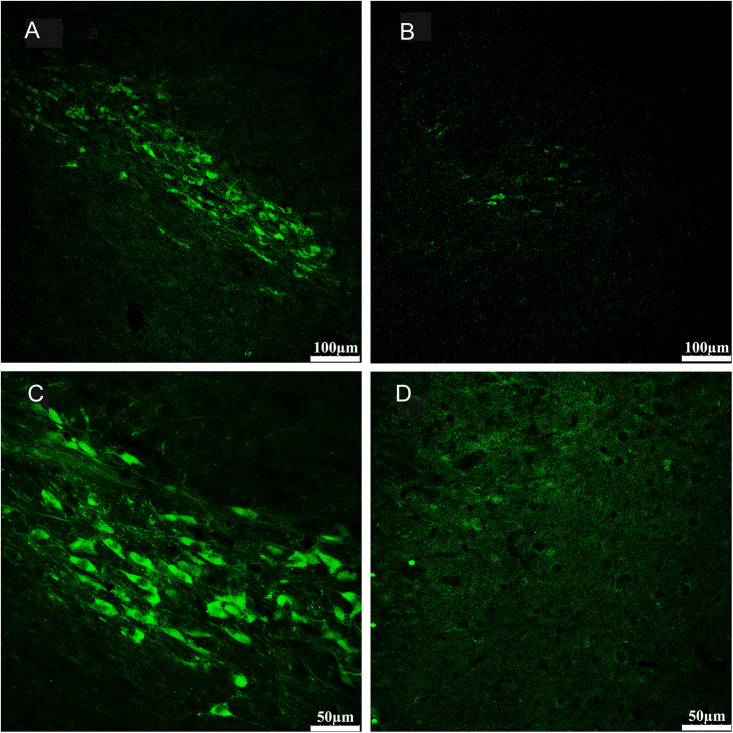
Sections from substantia nigra pars compacta of example rat showed fluorescence immunohistochemical staining for tyrosine hydroxylase (TH). Compared with the no- 6-OHDA-treated hemisphere **(A,C)**, which contained an intact population of dopaminergic neurons positive for TH staining, the 6-OHDA-treated hemisphere **(B,D)** displayed a severe loss of TH-positive neurons in the substantia nigra pars compacta. The subpanels in bottom **(C,D)** provide magnified views on the subpanels in top **(A,B)**, respectively. (**A,B** 100×; **C,D** 200×).

We compared the 6-OHDA-treated side with the contralateral non-6-OHDA-treated side in each PD rat (*n* = 11). The average percentage of TH-positive cells in the non-6-OHDA-treated hemispheres was considered 100%. The number of TH-positive neurons in the 6-OHDA-treated hemisphere was obviously decreased (mean loss of 92%, range 87–97%) compared with that in the contralateral non-lesioned hemisphere (Figure 3).

### Statistical Analysis

All results are expressed as the mean ± SEM. The threshold for significance was *p* < 0.05. The impact of 6-OHDA on DA cells was analyzed using an independent-samples *t*-test. Multiple comparisons were performed with two-way or repeated measures analysis of variance (ANOVA). *Post hoc* comparisons were made using Bonferroni’s test. Single comparisons were made using Student’s *t*-test for parametric data and the Mann–Whitney *U* test for non-parametric data. For phase-locked spike training, the phase angles between spikes and the LFP oscillations were analyzed using the Rayleigh test, and the mean vector length of groups of neurons was analyzed using the Mann–Whitney *U* tests for non-parametric data.

## Results

We recorded single units and LFPs from layer 5 of the M1 *in vivo* when animals were quiet, awake, and at rest or performing a forelimb-reaching task. High-quality extracellular activity data were isolated and recorded 2–3 weeks after electrode implantation surgery. Only the rats with lesions and those in which the tip of the electrode was histologically confirmed to be correctly positioned in layer 5 of the M1 were used for the statistical analyses. Ten rats were excluded; adequate turning behavior during apomorphine-induced rotation test was not shown by some of the rats (*n* = 2), the recording electrodes were misplaced or came off in other rats (*n* = 3), and some rats (*n* = 5) died during the experiment. Ultimately, 10 out of 37 rats were excluded, and a total of 27 rats were retained. Therefore, we analyzed extracellular activity (spiking and LFPs) in the PF in 6-OHDA-treated rats (*n* = 14) and control rats (*n* = 13).

### Characterization of Distinct Populations of M1 Neurons

**FIGURE 4:**
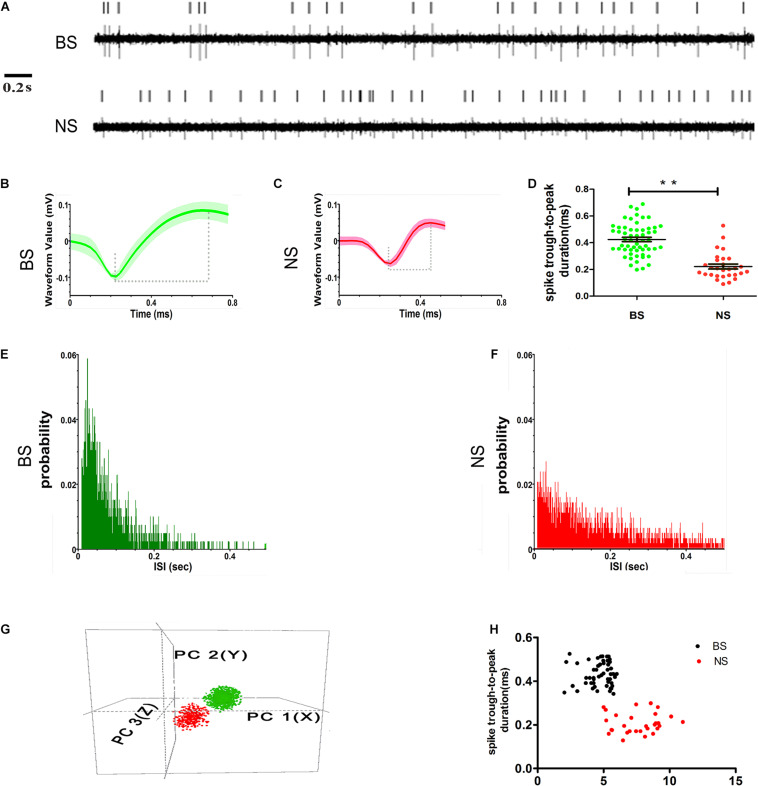
Examples of single units’ sorting technique and electrophysiological identification of BS (putative pyramidal neurons) and NS (putative interneurons) neurons in layer 5 of M1. Vertical lines represent single action potentials in spike training **(A)**. Electrophysiological identification in the high amplitude and long spike duration (trough-to-peak, between two dashed lines) waveforms of BS **(B)** and low amplitudes and short spike duration waveforms of NS **(C)**. The measurement in the two types of spike waveform durations (trough-to-peak) was different (** indicates *p* < 0.01) **(D)**. The ISI histograms show a clear refractory period, for the BS firing is characterized as a positively skewed distribution **(E)**. NS is characterized as relatively broad with a random distribution **(F)**. Isolated subpopulation neurons as distinct clusters in 3D space based on principal component (PC) analysis correspond to the BS with NS **(G)**. The scatter diagram plotting the duration versus baseline firing rate for all of the neurons recorded from control animals detected the BS with NS distinctly **(H)**.

The first step of this study was to distinguish whether there were subgroups of neurons in layer 5 of the M1 according to different single-unit properties in control rats. Neuronal activity in each channel was classified into disparate single units according to the widely accepted electrophysiological specificity of spike waveforms (Isomura et al., 2009; Chen et al., 2010; Kaufman et al., 2010, 2013). Based on these characteristics, putative pyramidal neurons and interneurons were readily identified. Pyramidal neurons generally exhibit unique spike waveforms that are distinct from those of interneurons. Overall, single units in our dataset were categorized into two groups: putative pyramidal neurons and putative interneurons (Figure 4). The very small number of neurons that had non-canonical waveforms (neither broad-spiking neurons nor narrow-spiking neurons) were not included in this analysis.

Neurons with high spike amplitudes and low spontaneous firing rates were defined as putative pyramidal neurons (Isomura et al., 2009; Kaufman et al., 2010; Reiner et al., 2010). Neurons with low spike amplitudes but a higher spontaneous firing rate were defined as putative interneurons (Barthó et al., 2004; Kaufman et al., 2010; Merchant et al., 2012). The putative pyramidal neurons exhibited broad spikes (BSs, green) width and asymmetric waveform, with both regular and burst-like firing patterns, typically showing intermittent grouped spikes separated by low-frequency tonic activities (Figures 4A,B), while the putative interneurons had narrow spikes (NSs, red) with lower spike amplitudes and a random firing pattern (Figures 4A,C). The spike duration, which was measured as the trough-to-peak duration, was significantly longer for BS neurons (0.42 ± 0.02 ms, *n* = 57), than for NS neurons (0.22 ± 0.02 ms, *n* = 29) (*p* < 0.001) in control animals (Figure 4D). The distribution of inter-spike intervals (ISIs) exhibited clear variations between the two subtypes. The distribution of the ISIs in BS neurons was characterized by an initial peak with a relatively narrow distribution, which indicates regular firing with bursts and pauses (Figure 4E), whereas the distribution of ISIs in the NS cells was characterized by a relatively broad and random distribution, which indicates irregular firing (Figure 4F). The BS and NS neurons could also be discriminated by comparing the 3D principal component clusters (Figure 4G). In addition, the difference between the BS and NS neurons could be seen in the scatter plot of spike duration against the baseline firing rate in control animals (Figure 4H).

### Effect of DA Loss on Spike Activity at Rest

We first examined all sorted M1 neurons in 6-OHDA-treated rats at rest. 6-OHDA differentially affected the firing properties of presumed pyramidal neurons and interneurons. Finally, we collected 83 neurons from the 6-OHDA-treated rats (*n* = 14); 55 were putative pyramidal neurons (BSs) and 28 were interneurons. Of the 86 neurons collected in the control rats (*n* = 13), 57 were pyramidal neurons and 29 were interneurons.

**FIGURE 5:**
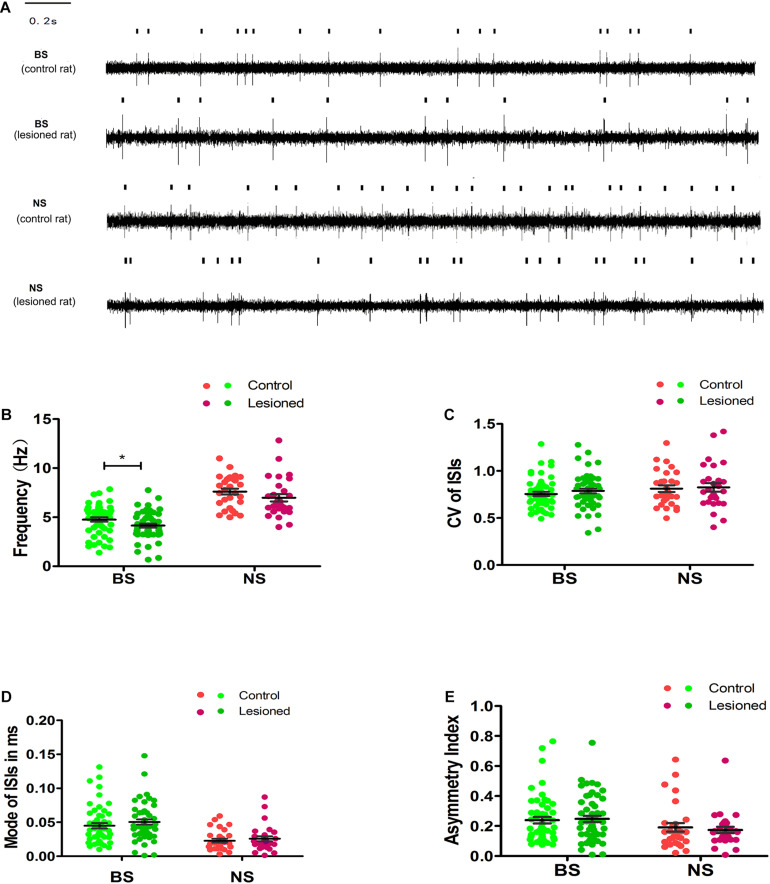
Illustrations of the Unit activity comparison of various parameters between distinct neuron-BS (putative pyramidal neurons) and NS (putative interneurons) from layer 5 of M1 in control and 6-OHDA-treated rats at rest. Representative raw recording of ongoing spikes of identified BS and NS neurons in control and 6-OHDA-treated rats **(A)**. The dots in the scatter plot showed the mean firing rate for each neuron from BS (green), with light green dots for control rats and dark green dots for 6-OHDA-treated rats, and from NS (red) neurons, with light red dots for control rats and dark red dots for 6-OHDA-treated rats. The comparison between control and 6-OHDA-treated rats was shown in average firing rates **(B)**, CV value **(C)**, Mode value **(D)**, and asymmetry index **(E)**. Black lines represent mean SEM over all neurons. * Indicates *p* < 0.05.

By summing the spikes of all neurons within a group, we found that the population spike rates of the two groups in the M1 were different between 6-OHDA-treated rats and control rats. The mean firing rate of BS neurons in 6-OHDA-treated rats (4.15 ± 0.25 spikes/s, *n* = 55) was slightly but significantly reduced compared with that of BS neurons in control rats (4.76 ± 0.21 spikes/s, *n* = 57, *p* = 0.04). The spike rates of NS neurons remained unchanged between 6-OHDA-treated rats (6.98 ± 0.38 spikes/s, *n* = 28) and control rats (7.62 ± 0.30 spikes/s, *n* = 29, *p* = 0.19) (Figures 5A,B).

To further examine the 6-OHDA-induced effects on neuronal firing patterns, we analyzed the CV, mode, and asymmetry index. The resting firing pattern of the BS neurons was unchanged following 6-OHDA-induced lesion formation (Figures 5C–E) since, compared with the control, 6-OHDA had no effect on the CV (0.77 ± 0.02 vs. 0.76 ± 0.02, *p* = 0.346), the mode (0.05 ± 0.00 vs. 0.04 ± 0.00, *p* = 0.106), or the asymmetry index (0.25 ± 0.02 vs. 0.24 ± 0.02, *p* = 0.550).

There was no significant difference in the CV (0.83 ± 0.05 vs. 0.81 ± 0.05, 0.81 ± 0.04, *p* = 0.836) (Figure 5C), the mode (0.03 ± 0.00 vs. 0.02 ± 0.00, *p* = 0.463, Figure 5D), or the asymmetry index (0.17 ± 0.03 vs. 0.19 ± 0.02, *p* = 0.621, Figure 5E) in NS neurons in 6-OHDA-treated rats compared with control rats. In summary, these data indicate that the mean firing rate of BS neurons, but not NS neurons, was modestly reduced in 6-OHDA-treated rats.

### Effect of DA Loss on LFPs at Rest

**FIGURE 6:**
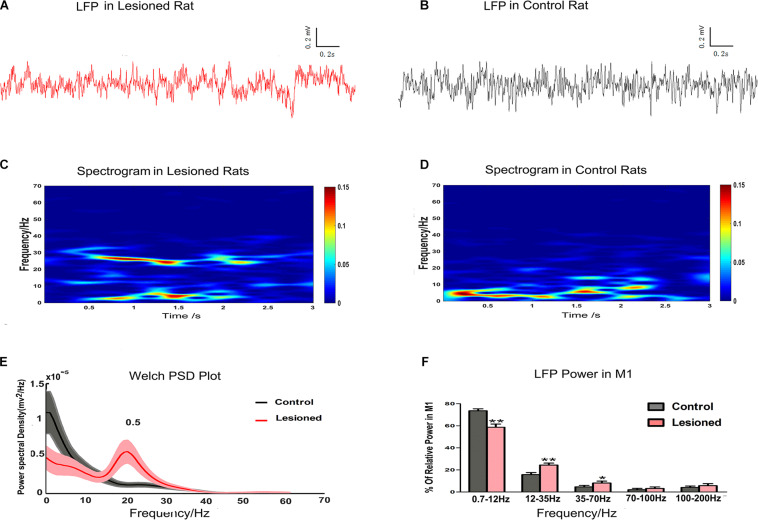
Illustration of the impact of DA cell loss on LFP activities at resting. Representative examples of raw LFP recordings in M1 at rest from a DA neuron 6-OHDA-treated rat (plotted in red trace, **A**) and control rat (plotted in black trace, **B**). Mean spectrograms of LFP power in M1 of depletion rats (*n* = 14 rats, **C**) and control rats (*n* = 13 rats, **D**). Data were averaged across all sessions. The color scale is logarithmic with respect to greater spectral power illustrated in red color. The frequency distribution of the LFP calculated by Welch estimation were average over all sessions in control (black line) vs. depletion rats (red line) **(E)**. Bar graphs showed the mean relative LFP power from different frequency bands in the control (black bars) and the depletion rats (red bars), which significantly decreased at 0.7–12 Hz and significantly increased at 12–35 and 35–70 Hz in the 6-OHDA-treated rats compared with the control rats **(F)**. Error bars represent SEM, * indicates *p* < 0.05 and ** indicates *p* < 0.01.

In this study, LFP data from the depletion rats (*n* = 14) and control rats (*n* = 13) were compared using the 3 s segments from at least 5 min of data recorded at rest. Examples of LFPs recorded in layer 5 of the M1 are shown for depletion rats (Figure 6A) and for control rats (Figure 6B). The LFP power spectra were present in the time-frequency spectrograms, with relatively distinct increasing and decreasing patterns in frequency bands. There were different LFP power frequency band distributions between the depletion rats (Figure 6C) and the control rats (Figure 6D). The LFP power spectra also showed obvious visible diversity in frequency band distributions between the depletion and control rats (Figure 6E). We subsequently computed the mean powers of distinct frequency bands in the 0.7–12, 12–35, 35–70, 70–100, and 100–200 Hz bands as a function across the entire signal band power (1–200 Hz, i.e., total power). Statistical differences (repeated-measures ANOVA) indicated distinct frequency band distributions between depletion and control rats. *Post hoc* comparison showed that the 0.7–12 Hz power was diminished in the depletion rats vs. the control rats (58.65 ± 2.89% vs. 73.51 ± 1.85%, *p* < 0.01), whereas the 12–35 Hz (24.34 ± 1.85% vs. 15.72 ± 1.86%, *p* < 0.01) and 35–70 Hz (8.09 ± 1.79% vs. 4.26 ± 1.35%, *p* < 0.05) powers were increased in the depletion rats vs. the control rats. There were no significant disparities in the 70–100 Hz and 100–200 Hz bands (Figure 6F).

### Effect of DA Loss on Movement-Related Firing Activity

Analysis of the proportions of cells indicated that most aspects of the reaching movements of the dopamine-depleted rats were significantly different from those of the control rats. The typical impacts of dopaminergic cell depletion on the M1 associated with reach-to-grasp movements were described in a previous study (Wang et al., 2016). Although the 6-OHDA-treated rats were able to grab food with their impaired paw, their level of correct performance of the task was evidently lower (49%, *n* = 14) than that of the control rats (90%, *n* = 13, *p* < 0.01). Unlike in control rats, reaching movement was fragmented by irregular trajectory of the limb. Due to an increase in the amount of time spent adjusting posture and grabbing food, the 6-OHDA-treated rats tended to exhibit slow performance when they used their preferred paw. The average movement duration (the time required for a rat to lift its paw off the ground and grab and retrieve a food pellet) was significantly longer for the dopamine-lesioned rats (1.25 ± 0.1 s, 14 rats) than for the control rats (0.96 ± 0.1 s, 13 rats, *p* < 0.01). In short, analysis of reaching movements demonstrated that rats with dopamine depletion had severe impairments in making voluntary movements and motor deficits similar to the slowness of movement (bradykinesia) observed in humans with PD.

To determine the effect of dopamine depletion on M1 activity in relation to specific grabbing movements, we analyzed alterations in distinct populations of neurons during individual reaches performed with the 6-OHDA-treated dominant paw. To this end, we constructed a peri-event raster plot and PETHs of firing rate aligned to grabbing food to detect modulations in forelimb movement in grasping food.

**FIGURE 7:**
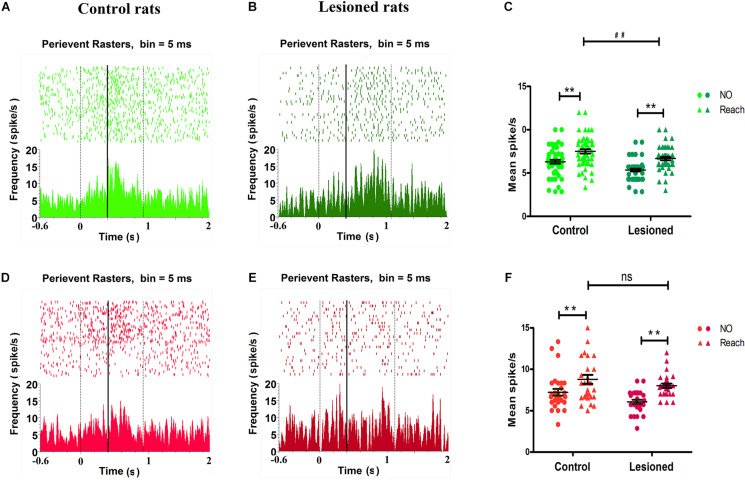
Impact of DA cell loss on different M1 type neurons activity in reaching movement. Illustrative examples of peri-event raster plots (top) and PSTHs (bottom) during baseline and movement in control rats **(A,D)** and in 6-OHDA-treated rats **(B,E)** for spike firing activities in BS (light green for control rat, dark green for 6-OHDA-treated rat) and NS (light red for control rat, dark red for 6-OHDA-treated rat), respectively. The accumulated histogram of spike activity (spike/s, bin width 5 ms) across trials is shown below each raster plot. The time epochs of successful reach were limited to the two black vertical dotted lines. The black vertical solid line indicated the time that rat handled limb advancement and broke the infrared ray, time = 0 indicated the point of time when the rat just lifted its paw. The basal line (background) is the time from –0.6 s to 0. The dots and trigons of scatter plot represent the average firing rate for each neuron in control (left) rats and depletion (right) rats **(C,F)**. BS showed their firing rates’ modulation difference for control rats (light green, *n* = 57 neurons) and 6-OHDA-treated rats (dark green, *n* = 55 neurons) during background (Not reaching, dots) or catching movement (Reaching, trigons) **(C)**. NS showed their firing rates’ modulation difference for control rats (light red, *n* = 29 neurons) and 6-OHDA-treated rats (dark red, *n* = 28 neurons) during background (Not reaching, dots) or catching movement (Reaching, trigons) **(D)**. **(C,F)** Show data comparing control and 6-OHDA-treated groups during not catching and reaching behavioral tasks, ** indicates *p* < 0.01, for effects of behavior or background, and ## indicates *p* < 0.01 for effects of behavior between control and 6-OHDA-treated rats, respectively. Black lines represent grand mean SEM over all neurons.

Analysis of the firing rate revealed that a distinct population of neurons in the M1 showed distinct modulations. As illustrated by the peri-event raster plot and PETHs, the forelimb reaching movement-induced changes in the firing of M1 neurons (between the two black vertical dotted lines) compared with the firing of M1 neurons before movement (before the first vertical dotted line) were cell type-selective (Figure 7). Between the period before movement and the movement period (i.e., background and successful reach), the firing activity of putative pyramidal neurons or BS neurons obviously varied with time, with activity being increased around the mark made by the infrared beam (black vertical solid line), which indicated the firing rates were substantially different during movement and before movement in both control rats (Figure 7A) and 6-OHDA-treated rats (Figure 7B). The 6-OHDA-treated rats exhibited modulation of action potentials, as indicated by reduced raster and activity amplitude compared with the control rats. To further statistically assess the effect of dopamine lesions on changes in the mean firing rate of BS neurons during movement, two-way ANOVA with group (control or 6-OHDA-treated) and behavior (background or reaching) as factors revealed significant influences on group [*F*(1,220) = 22.45, *p* = 0.00] and behavior [*F*(1, 220) = 44.27, *p* = 0.00] but no group × behavior interaction. The average firing rate during food-reaching movement (7.43 ± 0.23 spikes/s, *n* = 57) was enhanced compared with that during background behavior (6.29 ± 0.21 spikes/s, *p* = 0.01) in control rats. The same result was found in PD rats, as the mean firing rate during reaching (6.64 ± 0.11 spikes/s, *n* = 55) was enhanced compared with that during background behavior (5.33 ± 0.15 spikes/s, *p* = 0.01). These changes could prompt BS neuron spiking during reach-related activity. However, the firing rates of 6-OHDA-treated rats (6.64 ± 0.11 spikes/s, *n* = 55) were significantly lower than those of control rats (7.43 ± 0.23 spikes/s, *n* = 57, *p* = 0.03) during reaching. Together, these data demonstrated that the average firing rate of BS neurons increased during the transition in behavior state from background to movement and that dopaminergic cell depletion was associated with decreased firing rate of BS neurons when the animals were engaged in skilled forelimb food-reaching movement (Figure 7C).

Regarding putative interneurons, or NS neurons, there were changes in the firing rate during skilled forelimb movement in both control rats (Figure 7D) and 6-OHDA-treated rats (Figure 7E), as shown in the peri-event raster plot and PETHs. Two-way ANOVA with group and behavior as factors showed significant effects of group [*F*(1,110) = 6.13, *p* = 0.02] and behavior [*F*(1,110) = 20.11, *p* = 0.00] but no group × behavior interaction. The mean firing rat of NS neurons during food-reaching movement was obviously increased compared with that during background behavior in both the control group (8.78 ± 0.55 spikes/s, vs. 7.22 ± 0.41 spikes/s, *n* = 29, *p* = 0.03) and the 6-OHDA-treated group (8.00 ± 0.26 spikes/s, vs. 6.07 ± 0.25 spikes/s, *n* = 28, *p* = 0.00). These results indicate that NS neurons were also involved in reach-related activity. The firing rates of 6-OHDA-treated rats (8.78 ± 0.55 spikes/s, *n* = 29) were not significantly altered compared with those of the control rats (8.00 ± 0.26 spikes/s, *n* = 28, *p* = 0.21) during reaching (Figure 7F).

In summary, the impacts of 6-OHDA cytotoxicity on the forelimb reaching movement-related firing of M1 neurons were cell type-selective. Assessment of BS neuron action potentials revealed that dopamine depletion consistently decreased the mean firing rate of M1 neurons whether or not animals were engaged in highly skilled forelimb movement. Assessment of NS neuron action potentials in the 6-OHDA-treated rats revealed no significant alteration compared with those in control rats during skilled movement.

### Effect of DA Loss on Movement-Related LFPs

**FIGURE 8:**
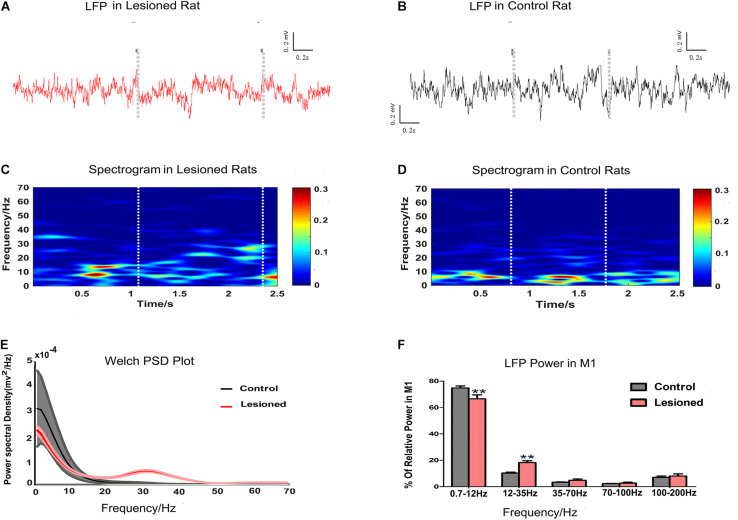
Illustration of the impact of DA cell loss on LFP activities during the grabbing food movement. Representative examples of raw LFP recordings in M1 during catching food, with the two gray vertical dotted lines representing the periods of successful reach from 6-OHDA-treated d rat (plotted in red trace, **A**) and control rat (plotted in black trace, **B**). With limitations in the two white vertical dotted lines indicating the periods of successful reach, mean spectrograms of LFP power show depletion rats (*n* = 14 rats, **C**) and control rats (*n* = 13 rats, **D**). The color scale is logarithmic with respect to greater spectral power illustrated in red color. The frequency distribution of the LFP calculated by Welch estimation were averaged over all sessions in control (black line, *n* = 13 rats) vs. depletion rats (red line, *n* = 14 rats) **(E)**. Bar graphs showed the LFP power from different frequency bands in the control (black bars) and the depletion rats (red bars), which significantly decreased at 0.7–12 Hz and significantly increased at 12–35 Hz in the 6-OHDA-treated rats compared with the control rats **(F)**. Error bars represent SEM, * indicates *p* < 0.05 and ** indicates *p* < 0.01.

Finally, we examined M1 LFP power spectra in each epoch of food-reaching movement using data extracted from each time window during repetitive successful reaches. M1 LFPs were enhanced or suppressed in the PD rats (*n* = 14) compared with the control rats (*n* = 13), as illustrated by the spectrogram plots (Figures 8A,B) and the Welch estimation frequency distribution in power spectra (Figure 8C,D). For each of the successful reaches, we calculated the mean powers in distinct frequency bands in the 0.7–12, 12–35, 35–70, 70–100, and 100–200 Hz bands as a function proportion to the entire signal band power (0.7–200 Hz, i.e., total power). Significant differences (repeated measures ANOVA) were revealed in distinct frequency bands. *Post hoc* comparison showed that the 0.7–12 Hz activity power was decreased in the PD rats compared with the control rats (66.65 ± 2.96 and 74.86 ± 1.5%, respectively, *p* < 0.01), while the 12–35 Hz activity power was increased in the PD rats (18.24 ± 1.47 and 10.35 ± 0.64%, respectively, *p* < 0.01). There were no significant changes in the 35–70, 70–100, and 100–200 Hz bands between the two groups (4.82 ± 0.98 vs. 3.41 ± 0.20%, 2.69 ± 0.64 vs. 2.31 ± 0.18% and 8.02 ± 1.67 vs. 7.07 ± 0.98%, respectively, *p* > 0.05, Figures 8E,F).

### Effect of DA Loss on the Relationship Between Spiking and LFPs

**FIGURE 9:**
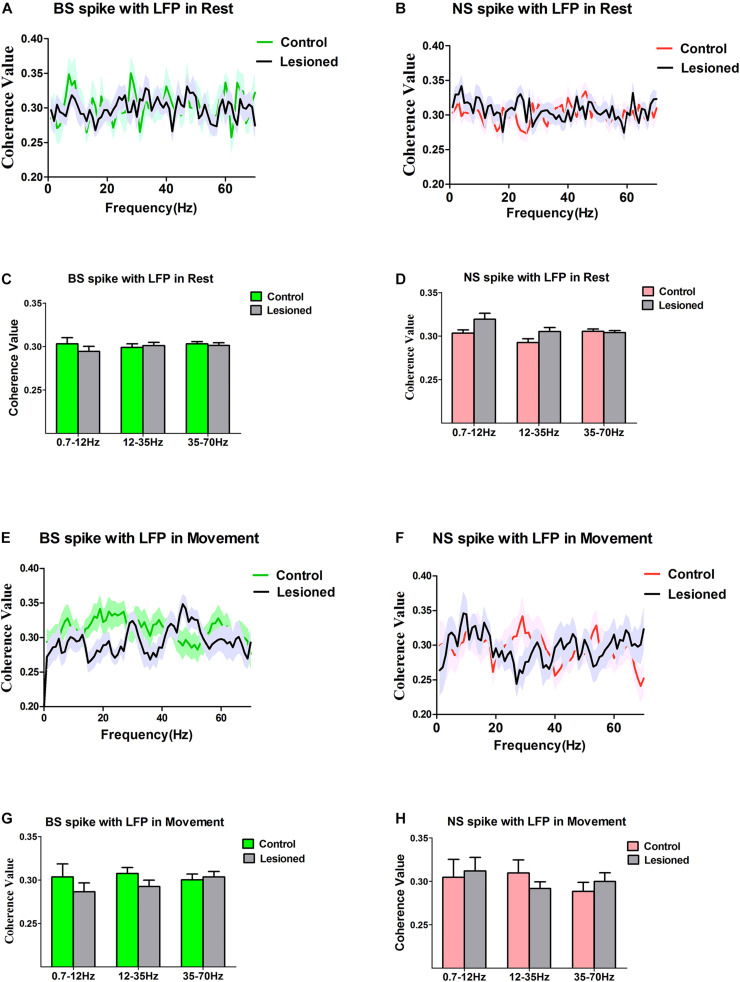
Characteristics of the Coherent values between the BS or NS neuron M1 spike activities with respect to LFP in dopamine-intact rats and 6-OHDA-treated rats. During the resting epoch, coherent values between BS (*n* = 31 neurons in control, 27 neurons in 6-OHDA-treated) and LFP activity at the 0.7–12 Hz, 12–35 Hz, and 35–75 Hz do not change significantly following dopamine cell lesion **(A,C)**, nor does it change in the NS (*n* = 26 neurons in control rats, 24 in 6-OHDA-treated rats) **(B,D)**. During the movement epoch, mean coherent values between BS spikes and LFP activity at the 0.7–12, 12–35, and 35–75 Hz exhibit no alteration in 6-OHDA-treated rats (*n* = 22 neurons) compared to the intact rats (*n* = 27 neurons) **(E,G)**, and there was no significant difference in the coherence between NS (*n* = 21 neurons in control, 23 in 6-OHDA-treated) spike-LFP pairs **(F,H)**.

**FIGURE 10:**
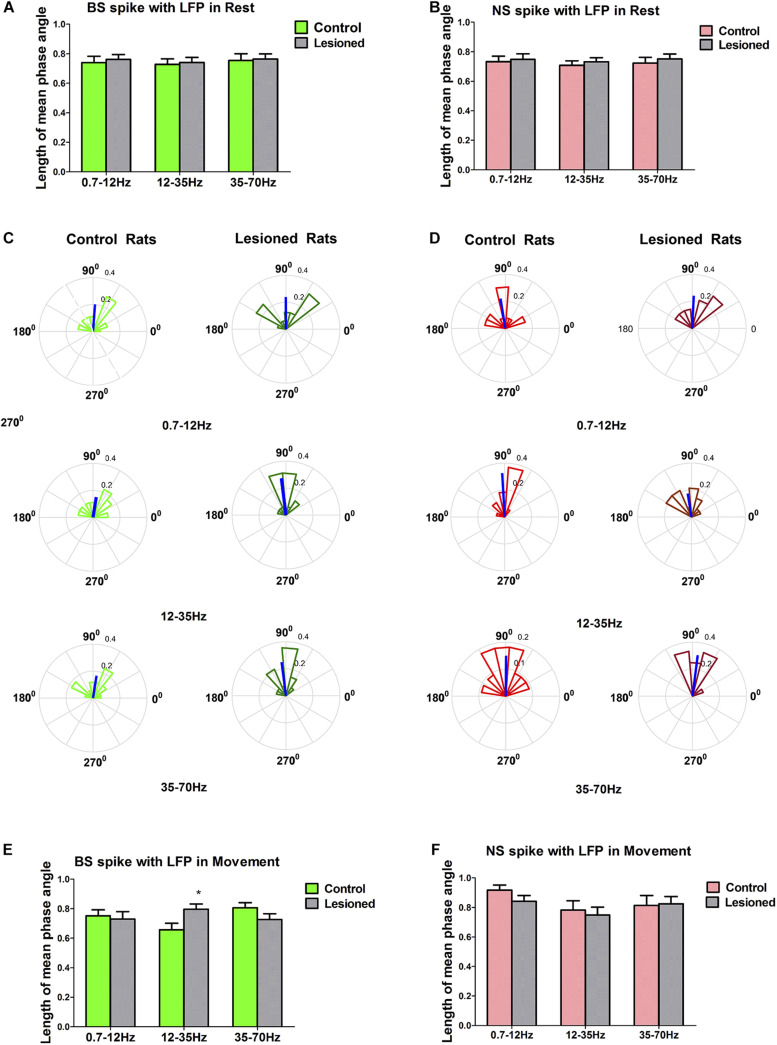
Characteristics of the phase locking between the BS or NS neuron M1 spike activities with respect to LFP in dopamine-intact and 6-OHDA-treated rats. During resting epoch, the phase locking levels represented by mean vector length between BS (*n* = 31 neurons in control, 27 neurons in 6-OHDA-treated) and LFP activity at the 0.7–12, 12–35, and 35–70 Hz does not change significantly following dopamine cell lesion **(A)**, nor does it change in the NS (*n* = 26 neurons in control, 24 in 6-OHDA-treated) **(B)**. During the movement epoch, phase circular plots show the distributions of phase relationships between BS or NS spikes and cortical LFPs at the 0.7–12, 12–35, and 35–70 Hz. Thick blue lines radiating from the center indicate a measure of the strength of concentration of the distribution of the mean phase angle of all BS (*n* = 22 neurons, 8 in 6-OHDA-treated rats; *n* = 27 neurons, 13 in control rats) or NS (*n* = 23 neurons, 7 in 6-OHDA-treated rats; *n* = 21 neurons 8 in control rats) spikes in that group. The mean angle of phase locking and greater vector lengths indicate lower variance in the distribution around the mean phase angle for each neuron. Note that BS neurons (dark green) in 6-OHDA-treated rats had significantly longer vectors and thus were more phase locked to LFP activities at 12–35 Hz than those neurons (light green) in control rats (*p < 0.05 Mann–Whitney *U* test) **(C,E)**. NS neurons (light red) in control rats did not significantly change in mean vector length compared to that of neurons (dark red) in 6-OHDA-treated rats at 0.7–12, 12–35, and 35–70 Hz **(D,F)**.

Finally, we investigated how the activity of individual M1 subgroup neurons varied over time with respect to ongoing cortical LFPs at different frequencies during rest or movement and whether these different neuronal subgroups have specific roles in shaping distinct LFPs in rats with chronic dopamine depletion. Special emphasis was placed on defining whether and how the action potentials of BS or NS neurons become entrained and excessively synchronized at 0.7–12, 12–35, and 35–70 Hz. These frequency ranges were chosen according to the above computed results, and alterations in LFPs in these three frequency bands were found between depletion and control rats. The relationship between BS or NS neuron spikes and LFPs was assessed using the spike-LFP coherence value (Figure 9) or the spike-LFP phase lock (Figure 10).

During the resting epoch, there was no change in the coherence between BS spiking and LFP oscillations following dopamine cell lesion, as the mean spike-LFP coherence value was not altered for BS neurons (*n* = 31 neurons from eight control rats vs. *n* = 27 neurons from seven 6-OHDA-treated rats) for LFPs at 0.7–12 Hz (0.30 ± 0.01 vs. 0.29 ± 0.01, *p* = 0.30), 12–35 Hz (0.30 ± 0.01 vs. 0.29 ± 0.01, *p* = 0.68), and 35–70 Hz (0.30 ± 0.01 vs. 0.30 ± 0.01, *p* = 0.70), as determined by the Mann–Whitney *U* test (Figures 9A,C), or for NS neurons (*n* = 26 neurons from seven control rats vs. *n* = 24 neurons from 13 6-OHDA-treated rats) at 0.7–12 Hz (0.30 ± 0.00 vs. 0.32 ± 0.01, *p* = 0.13), 12–35 Hz (0.29 ± 0.00 vs. 0.31 ± 0.01, *p* = 0.10), and 35–70 Hz (0.31 ± 0.01 vs. 0.30 ± 0.01, *p* = 0.72), as determined by the Mann–Whitney *U* test (Figures 9B,D). Together, these data confirm that during the resting epoch, both BS and NS neuron spikes showed no alterations that correlated with LFP oscillations.

During the movement epoch, whereas BS spike activity decreased, the mean coherence value between BS spiking and LFPs was not changed between 6-OHDA-treated rats (*n* = 22 neurons, eight rats) and control rats (*n* = 27 neurons, 13 rats) in the 12–35 Hz (0.31 ± 0.01 vs. 0.29 ± 0.01, *P* = 0.14), 0.7–12 Hz (0.30 ± 0.02 vs. 0.29 ± 0.01, *p* = 0.36) and 35–70 Hz ranges (0.30 ± 0.01 vs. 0.30 ± 0.01, *p* = 0.72), as determined by the Mann–Whitney *U* test (Figures 9E,G). The results did not show an effect of dopamine cell lesion on spike-LFP coherence in NS neurons (*n* = 21 neurons from eight control rats vs. *n* = 23 neurons from seven 6-OHDA-treated rats) at 0.7–12 Hz (0.30 ± 0.02 vs. 0.31 ± 0.02, *p* = 0.79), 12–35 Hz (0.31 ± 0.02 vs. 0.29 ± 0.01, *p* = 0.31), and 35–70 Hz (0.29 ± 0.01 vs. 0.30 ± 0.01, *p* = 0.43), as determined by the Mann–Whitney *U* test (Figures 9F,H). In summary, in the resting epoch, dopamine cell lesions induced no change in the coupling of BS spikes and LFPs during the reaching movement and had no significant effect on the relationship between spikes and LFPs in NS neurons.

Subsequently, to further examine the relationship between BS or NS time and cortical oscillations, we analyzed the phase-locking strength of BS or NS neuron spiking with respect to M1 LFPs according to the phase angle distribution of circular histogram plots to illustrate phase lock, i.e., the phase synchronism. The circular histogram plots for BS and NS neurons, which show the mean angle of phase locking and greater vector lengths, indicate lower variance in the distribution around the mean phase angle for each neuron.

During the resting epoch, the mean phase angles of firing of significantly phase-locked neurons (defined using Rayleigh’s uniformity test) in control and 6-OHDA-treated rats were not different, as indicated by the population vector lengths for each group of neurons. The mean vector length represented by a line radiating from the circle center for 6-OHDA-treated and control rats were similar in BS neurons in the 0.7–12 Hz (0.74 ± 0.04 vs. 0.76 ± 0.03, *p* = 0.82, Mann–Whitney *U* test), 12–35 Hz (0.73 ± 0.04 vs. 0.74 ± 0.03, *p* = 0.80), and 35–70 Hz ranges (0.75 ± 0.05 vs. 0.76 ± 0.04, *p* = 0.60) (Figure 10A), and were also similar in NS neurons in the 0.7–12 Hz (0.73 ± 0.04 vs. 0.75 ± 0.04, *p* = 0.71), 12–35 Hz (0.71 ± 0.03 vs. 0.73 ± 0.03, *p* = 0.57), and 35–70 Hz ranges (0.72 ± 0.04 vs. 0.75 ± 0.03, *p* = 0.59) (Figure 10B). Together, these data confirm that during the resting epoch, the firing of both BS and NS neurons in control rats and 6-OHDA-treated rats was not phase locked to LFP activity at 0.7–12, 12–35, and 35–70 Hz, with similar timing and precision.

During the movement epoch, the mean vector length in BS neurons (dark green) was significantly increased in 6-OHDA-treated rats (*n* = 22 neurons, eight rats) compared with that in neurons (light green) in control rats (*n* = 27 neurons, 13 rats) at 12–35 Hz (0.80 ± 0.04 vs. 0.66 ± 0.04, *p* = 0.032, Mann–Whitney *U* test), but was not changed at 0.7–12 Hz (0.73 ± 0.05 vs. 0.75 ± 0.04, *p* = 0.79) and 35–70 Hz (0.73 ± 0.03 vs. 0.81 ± 0.04, *p* = 0.09) (Figures 10C,E). In contrast to what was found for BS neurons, dopamine depletion had little impact on the mean vector length in NS neurons in 6-OHDA-treated rats (*n* = 23 neurons, seven rats) relative to control rats (*n* = 21 neurons, eight rats) at 0.7–12 Hz (0.92 ± 0.04 vs. 0.84 ± 0.04, *p* = 0.68), 12–35 Hz (0.78 ± 0.06 vs. 0.75 ± 0.05, *p* = 0.57) and 35–70 Hz (0.81 ± 0.06 vs. 0.83 ± 0.05, *p* = 0.90) (Figures 10D,F). In summary, dopamine cell lesions showed that BS neurons in particular fired in a more phase-locked manner in the 12–35 Hz range during the reaching movement, whereas NS neurons did not. It thus follows that these two cell types might make different contributions to the LFP changes in M1 activity dynamics that we observed in our simultaneous spike and LFP recordings.

## Discussion

Here, we provide behavioral and electrophysiological evidence for the effects of nigro-striatal dopamine depletion-induced hemi-parkinsonian on extracellular discharges within layer 5 of the M1 during resting and movement activities in rats. The results demonstrated that specific alterations in the temporal organization of electrical activity at the level of single neurons and small neuronal populations in the M1 were induced in the dopamine-depleted hemisphere in hemiparkinsonian rats. These alterations alter the contributions of the M1 to the generation and execution of voluntary limb movements and are likely to be important factors underlying the movement deficits in PD.

We found that following dopamine depletion, BS neurons (presumptive pyramidal neurons) showed reduced firing rates during both inattentive rest and reaching movement. However, NS neurons (presumptive interneurons) were not significantly affected by dopamine depletion.

Furthermore, dopaminergic cell loss was also involved in specific modulations of LFP frequencies, specifically decreases in frequency at 0.7–12 Hz and increases in frequency at 12–35 Hz and 35–70 Hz during rest and reaching movement, respectively. It is noteworthy that the alterations in the specific frequency of LFPs was also accompanied by increases in the phase synchronization of M1 spikes to LFPs in the 12–35 Hz range (beta-frequency oscillations), which excessively emerge in cortico-basal ganglia circuits after dopamine depletion during reaching movement. These divergent results suggest that different cortical cell types perform distinct functions and that not all neuronal subtypes are affected in the same way by the induction of parkinsonism (Pasquereau and Turner, 2011; Shepherd, 2013; Li et al., 2019). Together, these changes in neural activity at the single-cell or population level demonstrated that reduced dopamine function is involved in profound disruption of motor cortex activity (Brazhnik et al., 2012; Dejean et al., 2012; Shepherd, 2013; Pasquereau et al., 2016). Because pyramidal neurons in layer 5 of the M1 are the source of the strongest and most direct motor pathway from the cortex to the brain stem and the spinal cord involved in movement execution, the particular sensitivity of M1 BS neurons to the loss of dopamine may be an important contributor to the motor impairments in PD (Harris and Shepherd, 2015; Schnepel et al., 2015).

### Impact of Dopamine Depletion on Presumptive Pyramidal Neurons

A major finding of the current study is that putative pyramidal neurons in parkinsonism are associated with reduced firing rates at rest and in movement-related activity. These data are in partial agreement with a previous study in which animal PD models demonstrated significantly reduced movement-related activity in the M1. A study on a rodent acute drug-induced PD model revealed that dopamine-induced disruption of M1 activity was associated with a reduced baseline firing rate, bursting activity, and movement-related firing (Parr-Brownlie and Hyland, 2005). Studies on a primate chronic 6-OHDA-treated model reported various deficits during simple single-joint tasks, including reductions in mean spike incidence, the amplitude of responses (Watts and Mandir, 1992), and reduced reciprocal activity (Doudet et al., 1990), which is consistent with the hypothesis that pyramidal neurons are likely to be a central factor of the extent to which neural activity encodes movement parameters and play a crucial role in the pathophysiology of parkinsonism in monkeys (Pasquereau et al., 2016). This pyramidal neuron sensitivity could be explained by the relatively high density of direct dopamine input in the deep layers of the M1 from the ventral tegmental area (Berger et al., 1991) and the substantia nigra (Debeir et al., 2005), which are affected by degeneration of dopaminergic neurons in PD. Therefore, with regard to presumptive pyramidal neuron activity, the results confirmed the proposal made in classical pathophysiologic models of PD that increased activity in the basal ganglia output nuclei would induce decreased activity in cortical neurons (Nambu et al., 2015), which may be related to cortical reorganization in an attempt to compensate for the diminished basal ganglia cortical input (Guo et al., 2015; Xu et al., 2017). However, there is little consensus on whether mean firing rates in the M1 are changed in the parkinsonian state. Some research on parkinsonian animals failed to find modifications in the firing rates of M1 neurons (Goldberg et al., 2002) or revealed only transient changes (Dejean et al., 2012). Several experimental factors may have contributed to the different results regarding M1 mean firing rates in the parkinsonian state. One example is the diversity of cortical neurons, which are diverse in morphology, cluster or laminar distribution, neurochemical synaptic transmission, and interconnectivity. These inconsistencies might also be explained by discrepancies between the tasks used to control behavioral states (e.g., simple or skill movement) (Watts and Mandir, 1992; Pasquereau et al., 2016) in different studies and the brain states under which the studies were conducted (e.g., awake or under anesthesia) (Brazhnik et al., 2012; Li et al., 2012; Rios et al., 2019).

### Impact of Dopamine Depletion on Presumptive Interneurons

The second major finding of the current study is that presumptive interneurons and NS neurons in the M1 also exhibit temporal differences in the activation of self-initiation of voluntary forelimb movement. Notably, although there were firing frequency alterations before and after reaching movement, there were no significant differences in the firing rate of NS neurons in 6-OHDA-treated rats compared with control rats. These results suggest that NS neurons may be specifically differentiated to modulate motor information after dopamine cell lesioning. This implies that NS neurons are probably responsible for the ongoing modulation of command-like activity of presumptive pyramidal neurons during the execution of a single voluntary movement. Therefore, we cannot exclude the possibility that the results were merely due to the simplicity of the behavioral task and the rough data analysis. It seems most likely that NS neurons achieve temporally equipollent or homeostasis of feedforward and feedback inhibition to maintain stable function (Turrigiano, 2011; Hengen et al., 2016; Naka and Adesnik, 2016; Ma et al., 2019). Although the interneurons in the M1 are a minority cell type, their diversity and dense axonal arborization allow them to accommodate numerous cortical functions and behaviors through feedforward and feedback inhibition, not only from intralaminar circuits but also from other cortical layers in the local circuit (Isaacson and Scanziani, 2011; Apicella et al., 2012; Pluta et al., 2015; Hengen et al., 2016; Naka and Adesnik, 2016). On the other hand, this result seems to suggest that these interneurons represent a smaller population that has a smaller correlation with dysfunction in this behavior or process in the functional decompensatory period after dopamine cell lesioning (Turrigiano, 2011). Thus, our observation supports the idea that interneurons may underlie command shaping or balancing recurrent information rather than command gating or alternating temporal information in the M1 (Isomura et al., 2009). Unfortunately, to our knowledge, there is still a lack of understanding of how interneuron activity contributes to the cortical functions and behaviors disturbed in parkinsonism. The existing evidence on whether the baseline activity of putative interneurons is reduced (Alam et al., 2017) or elevated in parkinsonism is mixed (Brazhnik et al., 2012). This discrepancy may be explained by the fact that neuronal spike output is driven by the integration of synaptic inputs rather than depolarizing current steps from transient synaptic conductances (Isaacson and Scanziani, 2011; Turrigiano, 2011). Under different physiological or pathological conditions, with the variational modification of abnormal synaptic plasticity, most responses of the interneurons would be constrained by the state of the local network and not by behavioral contingencies (Kepecs and Fishell, 2014; Guo et al., 2015).

### Impact of Dopamine Depletion on Local Field Potentials

This study confirmed that the parkinsonian state is associated with substantially more prominent variation in LFP power than single spike activities, i.e., an increase in LFP power at 12–35 Hz and a decrease in LFP power at 0.7–12 Hz both during food-reaching movements and at rest. The results support the hypothesis that oscillations at 12–35 Hz (the beta frequency range) are linked to neural patterns with slow onset of new voluntary movements (Gilbertson et al., 2005), while the relationship between absolute beta levels and concurrent clinical state in PD is less clear (Galvan et al., 2015; Wang et al., 2015; West et al., 2018; Cagnan et al., 2019). It is also unclear whether the M1 activity changes induced by 6-OHDA are a consequence (via inherent neural feedback to the M1) or a cause (via attenuation of efferent motor commands) (West et al., 2018; Cagnan et al., 2019). However, some studies have shown that beta frequency is strongly correlated with rigidity and bradykinesia in PD (Chen et al., 2010; Little et al., 2012) and is a target of therapeutic interventions that minimize beta frequencies (Qasim and Jacobs, 2016). In addition, using closed loop stimulation, akinesia and bradykinesia appeared to be related to oscillatory activity, not firing rate, critically supporting the concept that low-frequency oscillations (around beta frequencies) play a key role in a non-human primate model of PD (Johnson et al., 2016). Our study did not directly test this theory, but the results indicated that a parkinsonian state of elevated power at 12–35 Hz occurs naturally at rest and during the performance of a specific behavioral task. These findings led to the idea that the pathophysiology of PD movement disorders may correlate not only with alterations in the firing rates of M1 neurons but also with abnormal large-scale neuronal population oscillations (Wang et al., 2015; Geng et al., 2016a, 2019). Based on current evidence that dopamine depletion generates more prominent variation in LFPs than in spikes, it seems reasonable to consider that the altered dynamics observed in PD are not only pathological causatively but rather associated with a loss of DA driven by upstream inputs from the basal ganglia-thalamocortical circuit (Surmeier et al., 2007; Nakamura et al., 2014).

### Impact of Dopamine Depletion on the Spike-LFP Relationship

In addition to single neurons or LFPs, the abnormal expression of synchronized activity between single-neuron spiking and LFP in the beta frequency band (12–35 Hz) has increasingly been used to explain the pathophysiology of parkinsonian in the brain (Walters et al., 2007; Mallet et al., 2019; Zhang et al., 2019). Our data indicate that following chronic dopamine depletion, which occurs in parkinsonian rats, BS neurons in the M1 not only fired at abnormally reduced spike rates and exhibited abnormal LFP oscillations but were particularly prone to exhibiting aberrant phase-locked firing to ongoing cortical LFP oscillations, preferentially recruited to exaggerated 12–35 Hz (beta frequency) during reaching movement. The data showed that BS neurons are particularly prone to being recruited to abnormal network oscillations, which provides insight into the excessive beta oscillations in the M1 that can guide strategies to interfere with their generation and maintenance in parkinsonism (Brazhnik et al., 2014; Dupre et al., 2016). This observation is consistent with the hypothesis that cortical rhythms entrain basal ganglia activity more readily after loss of dopamine and suggests that feedback via thalamocortical projections may contribute to beta oscillations in the motor cortex (Brazhnik et al., 2016) and parallel feedback loops in the basal ganglia network, such as the globus pallidus-subthalamic nucleus circuit (Magill et al., 2000; Sharott et al., 2012; Mallet et al., 2019).

In addition, this study revealed that single-neuron-spiking in the M1 was only weakly synchronized to LFP oscillations. The spike-LFP coupling relationship measures revealed only putative pyramidal neurons in the M1 become synchronized with LFPs in beta oscillations during movement following chronic dopamine depletion. Conversely, putative interneurons are neither abnormally active nor synchronized to a large extent during ongoing cortical oscillations. Furthermore, the spike-LFP coherence values for both the BS and NS neuron spikes showed no significant differences during either rest or movement. Notably, considering that the pathological beta synchronization in the M1 resulting from dopamine lesioning is widely or heterogeneously distributed across the network and unable to be attributed to any individual structure (McGregor and Nelson, 2019), the current finding of a weak relationship between spikes and LFPs in the M1 suggests that cortical pyramidal neurons are not the main origins of pathological beta rhythm. Therefore, the current study suggests that the M1 neuronal type-selective spiking activity is positively correlated with the strength of phase locking to cortical beta oscillations in hemiparkinsonian rats, and beta synchronization is unlikely to be determined by individual neurons in the cortex but rather to be determined by different neuronal populations in the network, whether physiological, pathological, or compensatory (Vitrac and Benoit-Marand, 2017; Cagnan et al., 2019).

## Conclusion

Collectively, these data indicate that chronic dopamine depletion alters not only the firing rates of neurons in layer 5 of the M1 but also excessively aberrant LFP oscillations and synchronized LFPs at beta oscillation frequencies. Considering that primary efferent pathways that transmit motor information from the M1 to the spinal cord originate from pyramidal neurons, these findings demonstrate the possibility that dopamine cell loss affects spiking activity that a subset of pyramidal neurons is especially sensitive to these effects. Despite these findings, the LFP power frequency bands at 12–35 Hz are greatly enhanced in the parkinsonian state, which refines and expands the hypothesis that abnormal cortical local population activity is also an important component of the pathophysiological mechanisms of PD. Overall, despite the significance of this experimental study, it remains unclear whether the changes in neuronal signals in the parkinsonian state can be clearly linked with the emergence of parkinsonism. Therefore, further studies on pathological motor cortex activity in PD are needed.

## Data Availability Statement

The original contributions presented in the study are included in the article/Supplementary Material, further inquiries can be directed to the corresponding author/s.

## Ethics Statement

The animal study was reviewed and approved by Animal Ethics Committee of Shandong Normal University.

## Author Contributions

ML: formal analysis, performing experiments, data analysis, and writing original draft. XuW: formal analysis, data analysis, methodology, and software. XY: data curation, methodology, and software. XiW: data curation, validation, and resources. FC: data curation, validation, and resources. XZ: data curation, validation, and resources. SS: methodology, software, and data curation. FH: data curation and validation. QJ, MG, DC, YS, YL, QH, and ZZ: software and data analysis. MW: conceptualization, resources, supervision, funding acquisition, and writing review and editing. All authors contributed to the article and approved the submitted version.

## Conflict of Interest

The authors declare that the research was conducted in the absence of any commercial or financial relationships that could be construed as a potential conflict of interest.

## Footnotes

1 http://chronux.org
