# Supplementary material for: Roles of Motor Cortex Neuron Classes in Reach-Related Modulation for Hemiparkinsonian Rats
Source: Front Neurosci. 2021 Apr 27;15:645849. doi: 10.3389/fnins.2021.645849 (PMC8111217; doi:10.3389/fnins.2021.645849)
Supplement: Supplementary file 1 [file Data_Sheet_1.PDF]

Supplemental Table 1

Apomorphine-induced rotation test

Group	6-OHDA Treated (r/min) (Rats = 20)		Control (r/min) (Rats = 17)	
**Rats**	1	14	1	0.2
	2	26	2	0.1
	3	18	3	0.55
	4	26	4	0
	5	12	5	0
	6	13	6	0.3
	7	13	7	0.15
	8	11	8	0
	9	21	9	0.4
	10	13	10	0.25
	11	10	11	0.3
	12	9	12	0.5
	13	25	13	0
	14	10	14	0.15
	15	21	15	0.05
	16	18	16	0.25
	17	15	17	0
	18	9		
	19	20		
	20	16		

Supplemental Videos

Supplemental Video 1: The rats practice fetching food using their forepaw– control group;

Supplemental Video 2: The rats practice fetching food using their forepaw–6-OHDA group;

Supplemental Video 3: Apomorphine-induced rotation test – control group;

Supplemental Video 4: Apomorphine-induced rotation test –6-OHDA group.
